# Effect of ertugliflozin on left ventricular function in type 2 diabetes and pre-heart failure: the Ertu-GLS randomized clinical trial

**DOI:** 10.1186/s12933-024-02463-0

**Published:** 2024-10-22

**Authors:** Soo Lim, Jae Hyun Bae, Heran Oh, In-Chang Hwang, Yeonyee E. Yoon, Goo-Yeong Cho

**Affiliations:** 1 Division of Endocrinology and Metabolism, Department of Internal Medicine, Seoul National University Bundang Hospital, Seongnam, Republic of Korea; 2 Department of Internal Medicine, Seoul National University Hospital, Seoul, Republic of Korea; 3 Division of Cardiology, Department of Internal Medicine, Seoul National University Bundang Hospital, Seongnam, Republic of Korea

**Keywords:** Angiotensin-converting enzyme 2, Angiotensin (1–7), Diabetes mellitus, type 2, Ertugliflozin, Global longitudinal strain, Heart function tests, Sodium-glucose transporter 2 inhibitors

## Abstract

**Background:**

The therapeutic effects of ertugliflozin, a sodium-glucose cotransporter 2 inhibitor, on cardiovascular outcome are not fully understood. This study aimed to evaluate the efficacy and safety of ertugliflozin on cardiac function in people with type 2 diabetes and pre-heart failure.

**Methods:**

We conducted a 24-week randomized, double-blind, placebo-controlled trial involving individuals with type 2 diabetes inadequately controlled with antidiabetic medications. Participants with left ventricular hypertrophy, E/e’ >15, or impaired left ventricular global longitudinal strain (LVGLS) were randomized 1:1 to receive either ertugliflozin (5 mg once daily) or a placebo. The primary outcome was the change in LVGLS. Secondary outcomes included changes in left ventricular mass index (LVMI) and left ventricular ejection fraction (LVEF). Prespecified exploratory outcomes, including angiotensin-converting enzyme 2 (ACE2) and angiotensin (1–7) levels, were also assessed.

**Results:**

A total of 102 individuals (mean age, 63.9 ± 9.2 years; 38% women) were included. The ertugliflozin group showed a significant improvement in LVGLS (− 15.5 ± 3.1% to − 16.6 ± 2.8%, *P* = 0.004) compared to the placebo group (− 16.7 ± 2.7% to − 16.4 ± 2.6%, *P* = 0.509), with a significant between-group difference (*P* = 0.013). Improvements in LVMI and LVEF were also observed. Additionally, significant reductions in HbA_1c_, systolic blood pressure, whole-body and visceral fat, uric acid, proteinuria, N-terminal pro–B-type natriuretic peptide, and lipoprotein(a) were noted. ACE2 and angiotensin (1–7) levels significantly increased in the ertugliflozin group compared to the placebo group and correlated with changes in LVGLS [*r* = 0.456, *P* < 0.001 for ACE2; *r* = 0.541, *P* < 0.001 for angiotensin (1–7)]. Adverse events were similar between the two groups.

**Conclusions:**

This study demonstrated that ertugliflozin has beneficial effects on left ventricular function in individuals with type 2 diabetes and pre-heart failure, and it provided insights into potential underlying mechanisms.

**Clinical trial registration:**

ClinicalTrials.gov Identifier: NCT03717194.

**Supplementary Information:**

The online version contains supplementary material available at 10.1186/s12933-024-02463-0.

## Introduction

Heart failure (HF) represents a continuum of structural and functional cardiac impairments, where early detection and intervention are critical to preventing progression to advanced disease. Recent guidelines have redefined the concept of pre-heart failure (pre-HF) as a stage in which individuals have no prior or current symptoms of HF but exhibit evidence of structural heart changes, abnormal cardiac function, or elevated cardiac markers [1, 2]. The 2022 guidelines from the American Heart Association, American College of Cardiology, and Heart Failure Society of America classify pre-HF as stage B HF, characterized by structural heart disease—such as reduced ventricular systolic function, ventricular hypertrophy, increased filling pressures, or elevated levels of B-type natriuretic peptide or cardiac troponin—without symptoms of HF [1].

The prevalence of pre-HF in type 2 diabetes (T2D) is notably high. A community-based cohort study found that 58% of individuals with T2D and preserved ejection fraction (EF) had echocardiographic abnormalities consistent with pre-HF [3]. Despite being asymptomatic, these individuals are at increased risk of developing symptomatic HF, underscoring the need for early intervention. Moreover, people with T2D are often categorized as being at risk for HF [1].

Sodium-glucose cotransporter 2 (SGLT2) inhibitors, a class of antidiabetic medications, have demonstrated significant cardiovascular (CV) benefits, including reducing hospitalization for HF. CV outcome trials, such as EMPA-REG OUTCOME, CANVAS, DECLARE-TIMI 58, and VERTIS CV, have highlighted the efficacy of SGLT2 inhibitors in reducing HF-related outcomes in individuals with T2D [4–7]. However, current guidelines do not prioritize SGLT2 inhibitors for pre-HF, primarily due to limited evidence in this specific population [1, 8].

Understanding the cardioprotective mechanisms of SGLT2 inhibitors is essential, particularly for those in the pre-HF stage. While traditional measures like left ventricular ejection fraction (LVEF) have been used to assess cardiac function, left ventricular global longitudinal strain (LVGLS) has emerged as a more sensitive indicator. LVGLS can detect early cardiac dysfunction and offers superior prognostic value for CV mortality [9–12].

This study aims to investigate the effect of ertugliflozin, an SGLT2 inhibitor, on cardiac function in individuals with T2D and pre-HF. By focusing on LVGLS and other echocardiographic parameters, we intend to elucidate the potential mechanisms underlying the cardioprotective effects of ertugliflozin in this population.

## Methods

### Study design and participants

The Ertu-GLS study was a 24-week, single-center, randomized, double-blind, placebo-controlled, parallel-group trial (trial protocol is available in **Supplemental methods**). The study was approved by the Institutional Review Board of Seoul National University Bundang Hospital (SNUBH), Republic of Korea (B-1801-498-002), and conducted in accordance with the principles of the Declaration of Helsinki. Our trial outcomes are reported in line with the Consolidated Standards of Reporting Trials (CONSORT) 2010 statement and the CONSORT-Outcomes 2022 extension [13].

We enrolled individuals with T2D and pre-HF. The main inclusion criteria were as follows: 1) adults diagnosed with T2D who had been receiving treatment with one or more oral antidiabetic medications (such as metformin, sulfonylureas, thiazolidinediones, dipeptidyl peptidase-4 inhibitors, or alpha-glucosidase inhibitor, excluding SGLT2 inhibitors) and/or insulin according to local guidelines for at least 12 weeks without any dose adjustment before enrollment; 2) glycated hemoglobin (HbA_1c_) levels between 7.5% and 9.0% to avoid complications from additional glycemic control; 3) estimated glomerular filtration rate ≥45 mL/min/1.73 m^2^; 4) pre-HF, defined by at least one of the following criteria: left ventricular hypertrophy (LVH) with a left ventricular mass index (LVMI) ≥ 95 g/m^2^ in women or ≥ 115 g/m^2^ in men, E/e’ >15, or impaired LVGLS of exceeding − 16% [1].

Exclusion criteria included type 1 diabetes; HbA_1c_ > 9.5% or fasting plasma glucose (FPG) > 15.0 mmol/L (270 mg/dL) during screening; previous treatment with glucagon-like peptide-1 (GLP-1) receptor agonists within 12 weeks before screening; a history of diabetic ketoacidosis or hyperglycemic hyperosmolar state; systolic blood pressure (SBP) > 180 mmHg or diastolic blood pressure (DBP) > 95 mmHg; symptoms of HF; severe anemia; respiratory, hepatic, neurological, or psychiatric disorders; active malignant neoplasm; other major systemic disease; or systemic use of glucocorticoids for more than 10 consecutive days within 90 days prior to screening.

## Randomization and blinding procedures

After screening potential participants at the outpatient clinic of the Division of Endocrinology and Metabolism, Department of Internal Medicine, SNUBH, individuals who met the inclusion and exclusion criteria were enrolled in the study. To ensure adherence, participants were required to achieve at least 80% compliance with the prescribed medication regimen during the two-week period before randomization.

Randomization was conducted using a computer-generated sequence, ensuring that a 1:1 allocation to the two groups (ertugliflozin 5 mg or placebo). A permuted block design was used to maintain group balance throughout the trial. To further reduce bias, both participants and investigators were blinded to the treatment assignments. Participants were also blinded to their HbA_1c_ and FPG results to avoid unintentional unblinding. Additionally, urinary glucose excretion results, which could indicate SGLT2 inhibitor activity, were withheld to preserve blinding.

Echocardiographic, anthropometric, and biochemical parameters were assessed at baseline and week 24 in a blinded manner, ensuring that neither the assessors nor the participants knew the treatment group assignments.

If a participant’s FPG level exceeded 15.0 mmol/L (270 mg/dL) at the week 8 visit or 13.3 mmol/L (240 mg/dL) at the week 16 visit, the investigator could administer open-label rescue medications according to local standards of care. Any approved oral antidiabetic medications, except for other SGLT2 inhibitors, or insulin could be used to manage hyperglycemia. Even after initiating rescue therapy, the investigational product from the randomized phase was continued, maintaining the double-blind condition until the completion of the study.

## Study outcomes

The primary outcome was the change in LVGLS following treatment with ertugliflozin compared to placebo. Secondary outcomes included changes in LVMI, LVEF, E/e’, left atrial volume index (LAVI), and left ventricular end-diastolic volume (LVEDV). Prespecified exploratory outcomes examined the effects of ertugliflozin on whole body and abdominal fat mass or percentage compared to placebo. Biochemical parameters, such as HbA_1c_, lipid profiles, lipoprotein(a), uric acid, protein-to-creatinine ratio, high-sensitivity C-reactive protein (hsCRP), N-terminal pro–B-type natriuretic peptide (NT-proBNP), troponin T, and ketone bodies, were also evaluated. Additionally, we investigated changes in circulating levels of angiotensin-converting enzyme 2 (ACE2) and angiotensin (1–7) [14] as potential pathways through which SGLT2 inhibitors may reduce major adverse cardiac events. Safety outcomes included the assessment of all adverse events and treatment-emergent adverse events (TEAEs).

## Acquisition and analysis of transthoracic echocardiography

Two-dimensional (2D) transthoracic echocardiography was performed using commercially available ultrasound machines (Vivid 7 and Vivid E9, General Electric Medical Systems, Milwaukee, WI, USA). All measurements were conducted in a blinded manner throughout the study. Quantitative analysis of 2D echocardiograms followed standard guidelines [15].

For global 2D strain analysis, dedicated software (EchoPAC, GE, Vingmed, Norway) was used. The left ventricular (LV) endocardium was traced semiautomatically with manual adjustment in the apical 4-chamber, 3-chamber, and 2-chamber views. Peak strain was defined as the peak negative value on the strain curve during the cardiac cycle. Peak LVGLS was calculated as follows: LVGLS (%) = [*L* (end-systole) − *L* (end-diastole) / *L* (end-diastole) × 100] [16], where *L* represents the whole LV myocardium as a single large segment. LVGLS was averaged across the apical 4-chamber, 3-chamber, and 2-chamber views. As LVGLS reflects the longitudinal shortening of myocardial fibers and is expressed as negative values, they were converted to absolute|x| values for easier interpretation. LVGLS values closer to 0 indicated more impaired LV systolic function.

LV volume and EF were calculated using the modified Simpson biplane method, and LV mass was determined using the Devereux formula, then indexed to body surface area to obtain LVMI.

LV diastolic function was assessed using pulsed wave Doppler to measure peak early (E) and late diastolic mitral inflow velocities and deceleration time. Additionally, peak systolic velocities and early (e’) and late diastolic velocities at the septal mitral annulus were measured using tissue Doppler imaging. The E/e’ ratio was calculated to estimate LV filling pressure, with values exceeding 15 considered indicative of elevated LV filling pressure.

## Measurements of anthropometric and biochemical parameters

A detailed list of additional parameter measurements can be found in Supplemental methods and Table S1.

### Sample size calculation

The study hypothesizes that the test group (ertugliflozin) will show greater improvement in LVGLS at week 24 from baseline compared to the control group (placebo). As no previous studies had examined the effects of SGLT2 inhibitors on LVGLS at the time of trial design, we used data from studies assessing LV volume, a common marker for evaluating the impact of drug therapies on HF survival [17]. A clinically significant change of 10 mL in LV volume, equivalent to a 15% change in EF for individuals with pre-HF (EF ranging from 30 to 50%), was considered appropriate [18]. In HF populations, the standard deviation (SD) for the mean difference in LVEF has been reported as 7.5 [19]. Therefore, we conservatively estimated a baseline LVGLS of 15% in both the test and control groups. We expected a 15% improvement in LVGLS after ertugliflozin treatment, with no change after placebo treatment, and an SD of 3.75% for both groups. Accounting for an 18% of dropout rate, the minimum required sample size was calculated to be 100. We ultimately decided to recruit 102 participants (51 in each group) with 1:1 randomization.

### Statistical analysis

Continuous variables were presented as mean ± standard deviation, while categorical variables were expressed as numbers and percentages. Baseline characteristics were compared using Student’s *t*-test for continuous variables and the chi-square test for categorical variables. Paired *t*-tests were conducted to assess differences in echocardiographic and laboratory parameters between baseline and post-treatment measurements within each treatment group. A Student’s *t*-test was used to compare changes in parameters that show no significant baseline differences between the two groups. For echocardiographic parameters with significant or borderline differences at baseline, an Analysis of Covariance (ANCOVA) model, adjusted for baseline values, was employed to provide accurate estimates of treatment effects. Simple linear regression analyses were conducted to examine the relationship between changes in LVGLS and circulating levels of ACE2 and angiotensin (1–7). All *P*-values were two-sided, with statistical significance set at *P* < 0.05. Statistical analyses were performed using the IBM SPSS software, version 28.0 (Armonk, NY, USA).

## Results

### Baseline characteristics

**Fig. 1:**
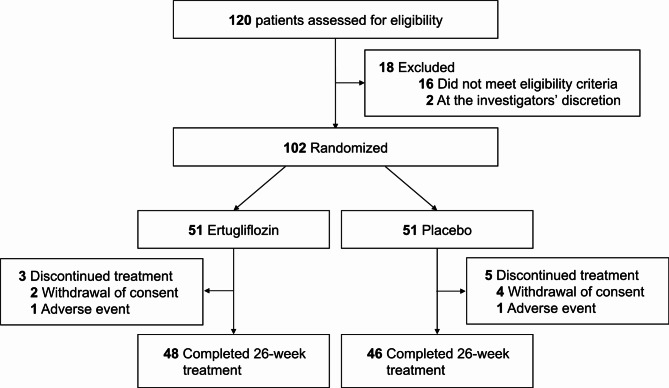
CONSORT diagram showing the trial profile

A total of 102 participants were enrolled in the study, with 51 individuals assigned to either the ertugliflozin or placebo groups (Fig. 1). Baseline characteristics are detailed in Table 1. The mean age was 62.5 years in the ertugliflozin group and 65.3 years in the placebo group. The mean body mass index (BMI) was 26.3 kg/m^2^ and 26.4 kg/m^2^, respectively. Initial HbA_1c_ levels were 8.3 ± 1.0% in the ertugliflozin group and 8.4 ± 1.1% in the placebo group. NT-proBNP levels were 153.1 ± 289.4 pg/mL and 166.2 ± 314.6 pg/mL, respectively. Baseline characteristics were well-balanced between the two groups, with no significant differences in comorbidities or medications, including angiotensin-converting enzyme inhibitors or angiotensin receptor blockers. Key echocardiographic parameters, such as LVGLS and LVEF, showed borderline non-significant differences between the groups (Table S2), while no significant differences were observed in the remaining parameters.

**Table 1:** Baseline characteristics of study participants

	Ertugliflozin (n = 51)	Placebo (n = 51)
	Mean ± SD	Mean ± SD
Age, years	62.5 ± 9.9	65.3 ± 8.4
BMI, kg/m^2^	26.3 ± 2.9	26.4 ± 3.6
SBP, mmHg	139.7 ± 16.3	140.6 ± 18.2
DBP, mmHg	78.8 ± 11.0	76.7 ± 8.2
Pulse rate, beat/min	78.5 ± 7.1	77.6 ± 10.8
HbA_1c_, %	8.3 ± 1.0	8.4 ± 1.1
FPG, mg/dL	143.4 ± 39.1	142.0 ± 37.7
PP2, mg/dL	272.4 ± 70.2	256.1 ± 68.9
Insulin, μU/ml	9.8 ± 4.1	9.5 ± 6.4
C-peptide, ng/ml	2.4 ± 1.6	2.4 ± 1.3
Total cholesterol, mg/dL	137.7 ± 27.6	143.6 ± 29.2
Triglyceride, mg/dL	148.3 ± 92.7	144.8 ± 74.8
HDL-cholesterol, mg/dL	46.0 ± 11.1	45.6 ± 10.1
LDL-cholesterol, mg/dL	73.1 ± 25.4	78.8 ± 23.6
AST, IU/L	28.9 ± 11.0	28.5 ± 10.4
ALT, IU/L	33.8 ± 19.6	31.2 ± 17.2
Creatinine, mg/dL	0.84 ± 0.22	0.81 ± 0.21
eGFR, mL/min/1.73 m^2^	90.1 ± 17.6	86.9 ± 16.8
Sodium, mmol/L	141.1 ± 2.3	140.5 ± 2.4
Potassium, mmol/L	4.5 ± 0.4	4.5 ± 0.3
Chloride, mmol/L	103.4 ± 4.3	103.5 ± 3.0
Uric acid, mg/dL	5.4 ± 1.3	5.2 ± 1.5
Urinary PCR, mg/g	349.3 ± 760.2	451.7 ± 1196.6
hsCRP, mg/L	0.13 ± 0.12	0.12 ± 0.14
NT-proBNP, pg/mL	153.1 ± 289.4	166.2 ± 314.6
*Comorbidity, n (%)*		
Hypertension	35 (68.6)	35 (68.6)
Dyslipidemia	48 (94.1)	41 (80.4)
*Medication, n (%)*		
Metformin	18 (35.3)	14 (27.5)
DPP-4 inhibitor	9 (17.6)	12 (23.5)
Sulfonylurea	12 (23.5)	8 (15.7)
Insulin	11 (21.6)	9 (17.6)
ACE inhibitor/ARB	16 (31.4)	17 (33.3)
Antiplatelet	10 (19.6)	9 (17.6)

ACE, angiotensin-converting enzyme; ALT, alanine aminotransferase; AST, aspartate aminotransferase; ARB, angiotensin receptor blocker; BMI, body mass index; DBP, diastolic blood pressure; DPP-4, dipeptidyl peptidase-4; eGFR, estimated glomerular filtration rate; FPG, fasting plasma glucose; HbA_1c_, glycated hemoglobin; HDL, high-density lipoprotein; hsCRP, high-sensitivity C-reactive protein; LDL, low-density lipoprotein; NT-proBNP, N-terminal pro–B-type natriuretic peptide; PCR, protein-to-creatinine ratio; PP2, 2-h postprandial glucose; SBP, systolic blood pressure; SD, standard deviation

### Changes in echocardiographic parameters

**Fig. 2:**
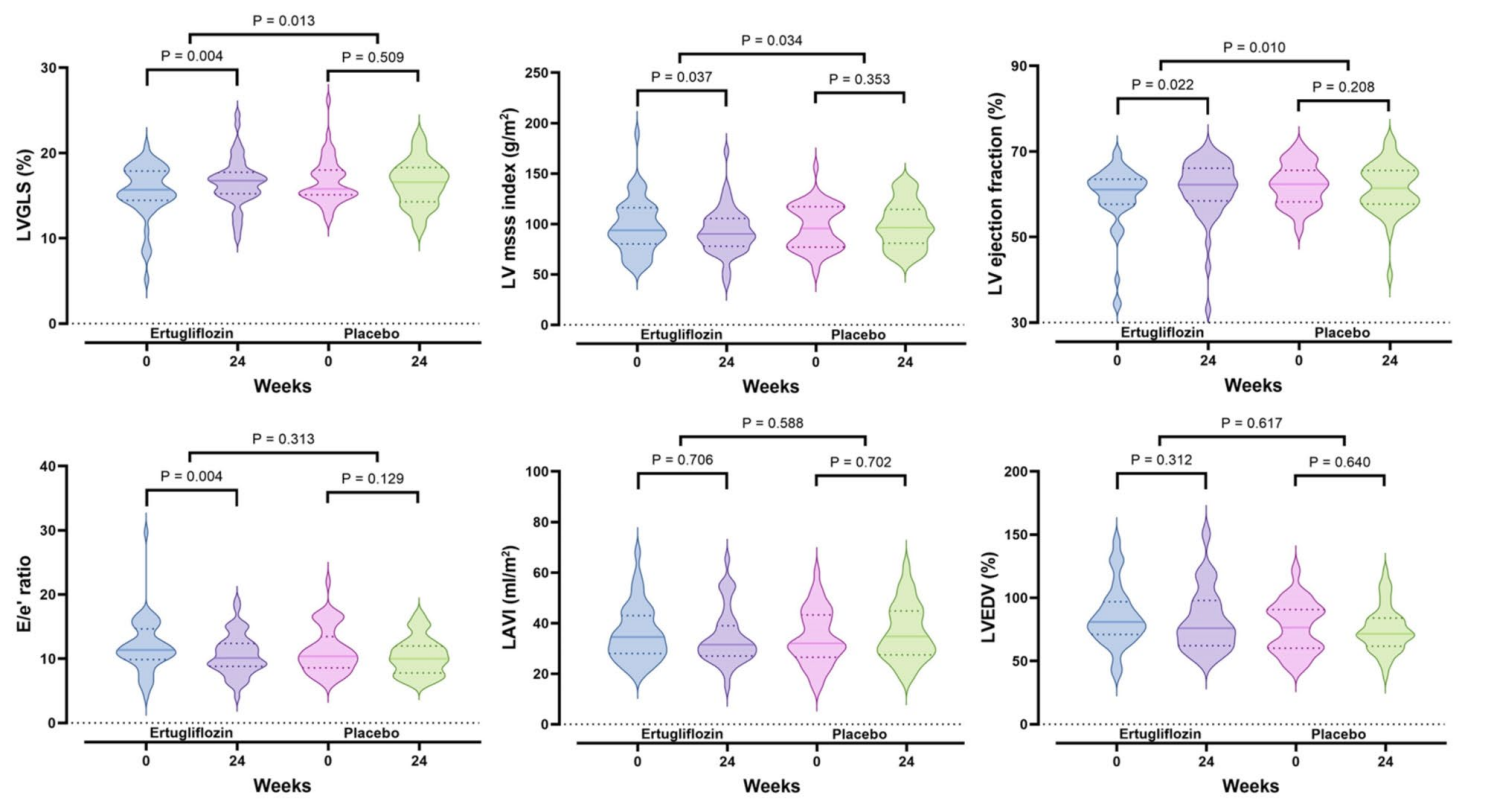
Comparison of changes in echocardiographic parameters between ertugliflozin treatment (*n* = 48) and placebo (*n* = 46) for 24 weeks. LAVI, left atrium volume index; LV, left ventricle; LVEDV, left ventricular end-diastolic volume; LVGLS, left ventricular global longitudinal strain. The dashed line represents the median, and the dotted lines indicate the quartiles

Figure 2 and Table S3 show the changes in echocardiographic parameters from baseline to week 24. The primary outcome, LVGLS, significantly improved in the ertugliflozin group, increasing from 15.5 ± 3.1% to 16.6 ± 2.8% (*P* = 0.004), with no significant change in the placebo group, which shifted from 16.7 ± 2.7% to 16.4 ± 2.6% (*P* = 0.509). This resulted in a significant difference between the two groups (*P* = 0.013) (Fig. 2A). LVMI significantly decreased in the ertugliflozin group (*P* = 0.037), with minimal change in the placebo group (*P* = 0.353), leading to a more pronounced in the ertugliflozin group compared to the placebo group (*P* = 0.034) (Fig. 2B). LVEF also showed significant improvement in the ertugliflozin group compared to the placebo group (*P* = 0.010) (Fig. 2C), aligning with the LVGLS results. For E/e’, there was a significant decrease in the ertugliflozin group (*P* = 0.004), with no substantial difference between the groups (Fig. 2D). LAVI and LVEDV showed no significant changes in either group, and there were no significant differences between the groups (Fig. 2E and F).

### Changes in anthropometric and biochemical parameters

At week 24, individuals in the ertugliflozin group showed significant reductions in BMI, body fat, abdominal visceral fat, and SBP compared to those in the placebo group. Although there was a noticeable decrease in DBP in the ertugliflozin group, it was not significantly different from the change observed in the placebo group (Table S4).

Laboratory tests demonstrated that ertugliflozin therapy led to significant improvements across several indices related to glycemic control, insulin resistance, and pancreatic beta-cell function compared to the placebo group (Table S4). Notably, in the ertugliflozin group HbA_1c_ decreased from 8.3 ± 1.0% to 7.7 ± 1.2% (*P* = 0.001), while no significant change was observed in the placebo group. Additionally, ertugliflozin therapy resulted in significant reductions in triglyceride, lipoprotein(a), uric acid, and proteinuria, as well as decreases in low-density lipoprotein (LDL) cholesterol and hsCRP levels, compared to the placebo group.

**Fig. 3:**
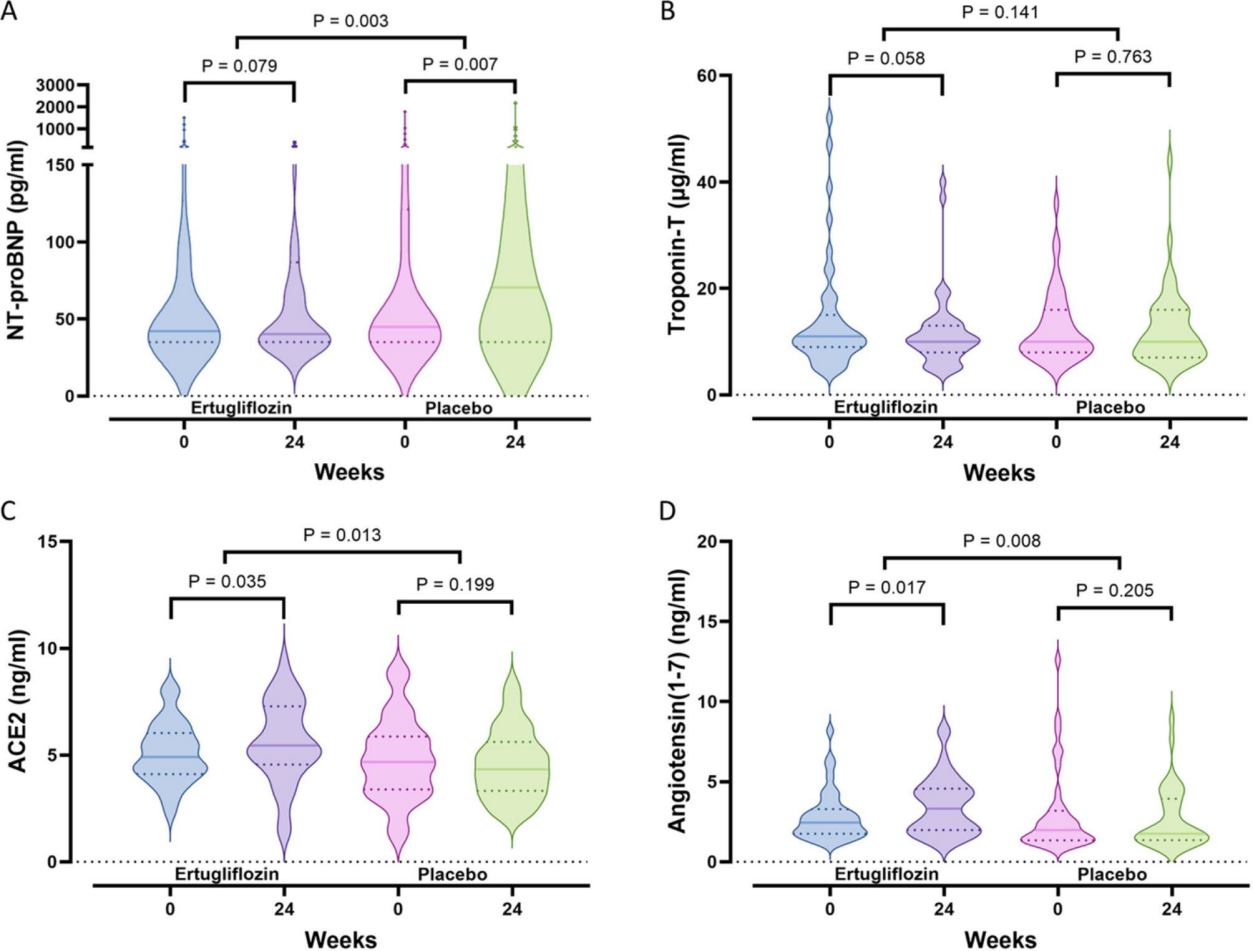
Comparison of changes in serum NT-proBNP (A), troponin-T (B), ACE2 (C), and angiotensin (1–7) (D) levels after 24 weeks of ertugliflozin treatment (*n* = 48) or placebo (*n* = 46). ACE2, angiotensin-converting enzyme 2; NT-proBNP, N-terminal pro–B-type natriuretic peptide. The dashed line represents the median, and the dotted lines indicate the quartiles

Other prespecified exploratory outcomes showed a significant decrease in NT-proBNP exclusively in the ertugliflozin group, resulting in a pronounced difference between the groups (*P* = 0.003). These changes were accompanied by a substantial increase in ketone bodies in the ertugliflozin group, along with a decrease in troponin-T levels, indicating reduced myocardial injury (Table S4 and Fig. 3A and B). Ertugliflozin also significantly increased ACE2 and angiotensin (1–7) levels, with no corresponding change in the placebo group, leading to a noteworthy difference between the groups (*P* = 0.013 and *P* = 0.008, respectively) (Table S4 and Fig. 3C and D). Interestingly, changes in LVGLS were positively correlated with alterations in ACE2 (*r* = 0.423, *P* < 0.001) and angiotensin (1–7) (*r* = 0.523, *P* < 0.001), as shown in partial correlation analyses controlling for age, BMI, SBP, and HbA_1c_.

### Safety

Throughout the study, adverse events were observed in 25.5% of participants in the ertugliflozin group and 23.5% in the placebo group, with no significant difference between the two groups. TEAEs occurred in 11.8% of the ertugliflozin group and 9.8% of the placebo group. Hypoglycemia was the most reported TEAE in the ertugliflozin group (5.9%), while urinary tract infections did not significantly differ between the groups. No serious adverse events were reported, and other adverse events were sporadic, with no significant differences between the groups (Table 2).

**Table 2:** Adverse events and treatment-emergent adverse events

	Ertugliflozin group (*n* = 51)	Placebo group (*n* = 51)	*P*
All adverse events, n (%)	13 (25.5)	12 (23.5)	> 0.999
Treatment-emergent adverse events, n (%)	6 (11.8)	5 (9.8)	> 0.999
Hypoglycemia	3 (5.9)	1 (2.0)	0.617
Severe hypoglycemia	0 (0)	0 (0)	–
Genital infection	2 (3.9)	1 (2.0)	> 0.999
Urinary tract infection	2 (3.9)	3 (5.9)	> 0.999
Dehydration	1 (2.0)	0 (0)	> 0.999
Amputation	0 (0)	0 (0)	–
Euglycemic ketoacidosis	0 (0)	0 (0)	–
Other adverse events, n (%)			
Gastrointestinal discomfort	3 (5.9)	3 (5.9)	> 0.999
Upper respiratory infection	2 (3.9)	3 (5.9)	> 0.999
Dizziness	3 (5.9)	1 (2.0)	0.617
Skin lesion	1 (2.0)	1 (2.0)	> 0.999
Allergy	2 (3.9)	3 (5.9)	> 0.999
Arthralgia or arthritis	2 (3.9)	3 (5.9)	> 0.999
Osteopenia	1 (2.0)	1 (2.0)	> 0.999
Glaucoma	0 (0)	1 (2.0)	> 0.999

## Discussion

In this study, ertugliflozin therapy significantly improved LVGLS in individuals with T2D and pre-HF. It also enhanced LVEF, reduced LVMI, and decreased hyperglycemia, insulin resistance, uric acid levels, proteinuria, and lipoprotein(a), BMI, body fat, and SBP compared to the placebo. Additionally, ertugliflozin increased ACE2 and angiotensin (1–7) levels, which positively correlated with changes in LVGLS.

The improvement in LVGLS is particularly notable as a marker for cardiac function in people with T2D during the early stages of HF. LVGLS, reflecting the deformation of the LV myocardium [20], provides better diagnostic and prognostic insights than traditional echocardiographic parameters, especially in those with preserved LVEF [21]. T2D is a known risk factor for HF, and impaired LVGLS significantly increases the risk of developing symptomatic HF, emphasizing the importance of assessing cardiac function in asymptomatic individuals with T2D.

Our study is among the first to demonstrate that ertugliflozin enhances LVGLS in this population, suggesting potential benefits in reducing CV events related to HF. LVGLS is valuable for detecting early cardiac dysfunction and evaluating treatment effectiveness, even in individuals with normal cardiac function by traditional measures [22, 23]. Ertugliflozin also reduce LVMI, consistent with findings for other SGLT2 inhibitors such as dapagliflozin and empagliflozin [9, 24]. Although the baseline LVMI was not notably high, its reduction is significant, as increased LVMI is associated with symptomatic HF and mortality [3]. The rise in LVEF in the ertugliflozin group further supports its positive impact on cardiac function, even in those with preserved LVEF.

In line with echocardiographic parameters, NT-proBNP levels significantly declined in the ertugliflozin group, indicating reduced myocardial stress [25]. This highlights that ertugliflozin may improve CV outcomes, including adverse events, hospitalization, and morality, beyond its effects on glycemic control [26–28].

Ertugliflozin was also found to increase ACE2 and angiotensin (1–7) levels, suggesting potential CV and renal benefits. These components of the nonclassical renin-angiotensin system may improve insulin sensitivity and protect pancreatic beta cells, offering additional metabolic advantages [29]. Ertugliflozin also reduced urinary protein excretion, a marker for HF risk [30], likely through pathways involving ACE2 and angiotensin (1–7) [14]. Additionally, ertugliflozin provided metabolic benefits, including lower levels of hsCRP, uric acid, and LDL cholesterol, indicating reduced inflammation and oxidative stress [31–34]. It also reduced lipoprotein(a) levels, which are associated with an increased risk of CV disease [35, 36].

This study has several limitations. First, the initial sample size calculation assumed a worse baseline LVGLS than observed. Additionally, a borderline but non-significant difference in baseline LVGLS between the ertugliflozin and placebo groups could raise concerns that regression to the mean might partly explain the improvements in the ertugliflozin group. While we cannot fully exclude this possibility, the improvement in LVGLS was statistically significant, clinically meaningful, and absent in the placebo group. Furthermore, LVMI decreased and LVEF increased significantly in the ertugliflozin group, reinforcing the improvements are unlikely to be solely due to regression to the mean. We also used an ANCOVA model to adjust for baseline differences in LVGLS, ensuring that the treatment effects were evaluated after accounting for any baseline imbalance. Second, although our population may have included individuals with HF with preserved EF, the low baseline NT-proBNP levels suggest that most participants were likely in a pre-HF state. Third, the relatively short duration of treatment exposure may have affected the results, though the benefits of SGLT2 inhibitors on HF outcomes are known to extend beyond glycemic control. Fourth, the study did not assess long-term clinical outcomes, such as HF hospitalization or CV mortality, indicating the need for longer follow-up studies. Finally, as this study was conducted within a Korean population, the findings may not be generalizable to other ethnic groups.

## Conclusions

In conclusion, ertugliflozin therapy significantly enhanced LV function and structure, as evidenced by improvements in LVGLS, LVMI, and LVEF, in individuals with T2D and pre-HF. It also resulted in favorable changes in ACE2, angiotensin (1–7), NT-proBNP, and lipoprotein(a) levels. Additionally, the therapy provided cardiometabolic benefits, including reductions in blood pressure, whole-body fat, and abdominal visceral fat, as well as improvements in glucose and lipid metabolism, and decreases in uric acid, proteinuria, and hsCRP. This study highlights novel mechanisms through which ertugliflozin may impact HF outcomes.

## Electronic supplementary material

Below is the link to the electronic supplementary material.

Supplementary Material 1

## Data Availability

No datasets were generated or analysed during the current study.

## Abbreviations

2D: Two dimensional
ACE2: Angiotensin-converting enzyme 2
ANCOVA: Analysis of Covariance
BMI: Body mass index
CANVAS: Canagliflozin Cardiovascular Assessment Study
CONSORT: Consolidated Standards of Reporting Trials
CV: Cardiovascular
DECLARE-TIMI 58: Dapagliflozin Effect on Cardiovascular Events–Thrombolysis in Myocardial Infarction 58
DBP: Diastolic blood pressure
EF: Ejection fraction
EMPA-REG OUTCOME: Empagliflozin Cardiovascular Outcome Event Trial in Type 2 Diabetes Mellitus Patients-Removing Excess Glucose
FPG: Fasting plasma glucose
GLP-1: Glucagon-like peptide-1
HbA_1c_: Glycated hemoglobin
HF: Heart failure
hsCRP: High-sensitivity C-reactive protein
LDL: Low-density lipoprotein
LAVI: Left atrial volume index
LV: Left ventricular
LVEF: Left ventricular ejection fraction
LVEDV: Left ventricular end-diastolic volume
LVGLS: Left ventricular global longitudinal strain
LVH: Left ventricular hypertrophy
LVMI: Left ventricular mass index
NT-proBNP: N-terminal pro–B-type natriuretic peptide
Pre-HF: Pre-heart failure
SBP: Systolic blood pressure
SD: Standard deviation
SGLT2: Sodium-glucose cotransporter 2
T2D: Type 2 diabetes
TEAE: Treatment-emergent adverse events
VERTIS CV: Ertugliflozin Efficacy and Safety Cardiovascular Outcomes trial
