# Supplementary material for: Effect of ertugliflozin on left ventricular function in type 2 diabetes and pre-heart failure: the Ertu-GLS randomized clinical trial
Source: Cardiovasc Diabetol. 2024 Oct 22;23:373. doi: 10.1186/s12933-024-02463-0 (PMC11515769; doi:10.1186/s12933-024-02463-0)
Supplement: Supplementary file 1 — Supplementary Material 1 [file 12933_2024_2463_MOESM1_ESM.docx]

**Supplemental methods**

**Ertu-GLS Randomized Clinical Trial Protocol** (pages 2–15)

**Measurement of biochemical parameters** (page 16)

**Ertu-GLS Randomized Clinical Trial Protocol**

1. **Objectives**

The aim of this study is to investigate the beneficial effects of ertugliflozin, a novel sodium-glucose cotransporter 2 (SGLT2) inhibitor, on cardiac function by assessing global longitudinal strain (GLS) and other hemodynamic parameters through echocardiography in individuals with type 2 diabetes (T2D) and pre-heart failure (pre-HF)

1. **Hypothesis**

We hypothesize that ertugliflozin will improve cardiac function in individuals with T2D and pre-heart failure.

1. **Study design**

This randomized, double-blind, placebo-controlled, parallel-group trial aims to enroll 120 participants. Individuals with T2D who have been on pharmacological treatment for at least 12 weeks, without any dose adjustments prior to enrollment, will be eligible for screening. The screening period will last up to two weeks before randomization. To qualify for randomization, participants must meet an 80% compliance rate, based on pill count, and the investigator's discretion during the run-in phase. After randomization, participants will be blinded to their glycated hemoglobin (HbA1c) and fasting plasma glucose (FPG) results. To maintain blinding, urinary glucose excretion (UGE) results will also be masked from participants. Urine glucose, albumin, calcium, and creatinine will be measured separately during on-site visits.

1. **Study participants**
   1. **Inclusion criteria**

- Individuals with T2D on stable oral antidiabetic medications, excluding SGLT2 inhibitors, for at least 12 weeks prior to enrollment, with no dose adjustments
- Estimated glomerular filtration rate (eGFR) ≥45 mL/min/1.73 m²
- Pre-heart failure (pre-HF), or stage B heart failure (HF), identified based on structural or functional markers (Fig. 1) [1, 2]:
  - Structural abnormality defined as moderate left ventricular hypertrophy (LVH), indicated by a left ventricular mass index (LVMI) ≥95 g/m² in women or ≥115 g/m² in men
  - Functional impairment defined as E/e’ > 15 and/or left ventricular global longitudinal strain (LVGLS) > −16%


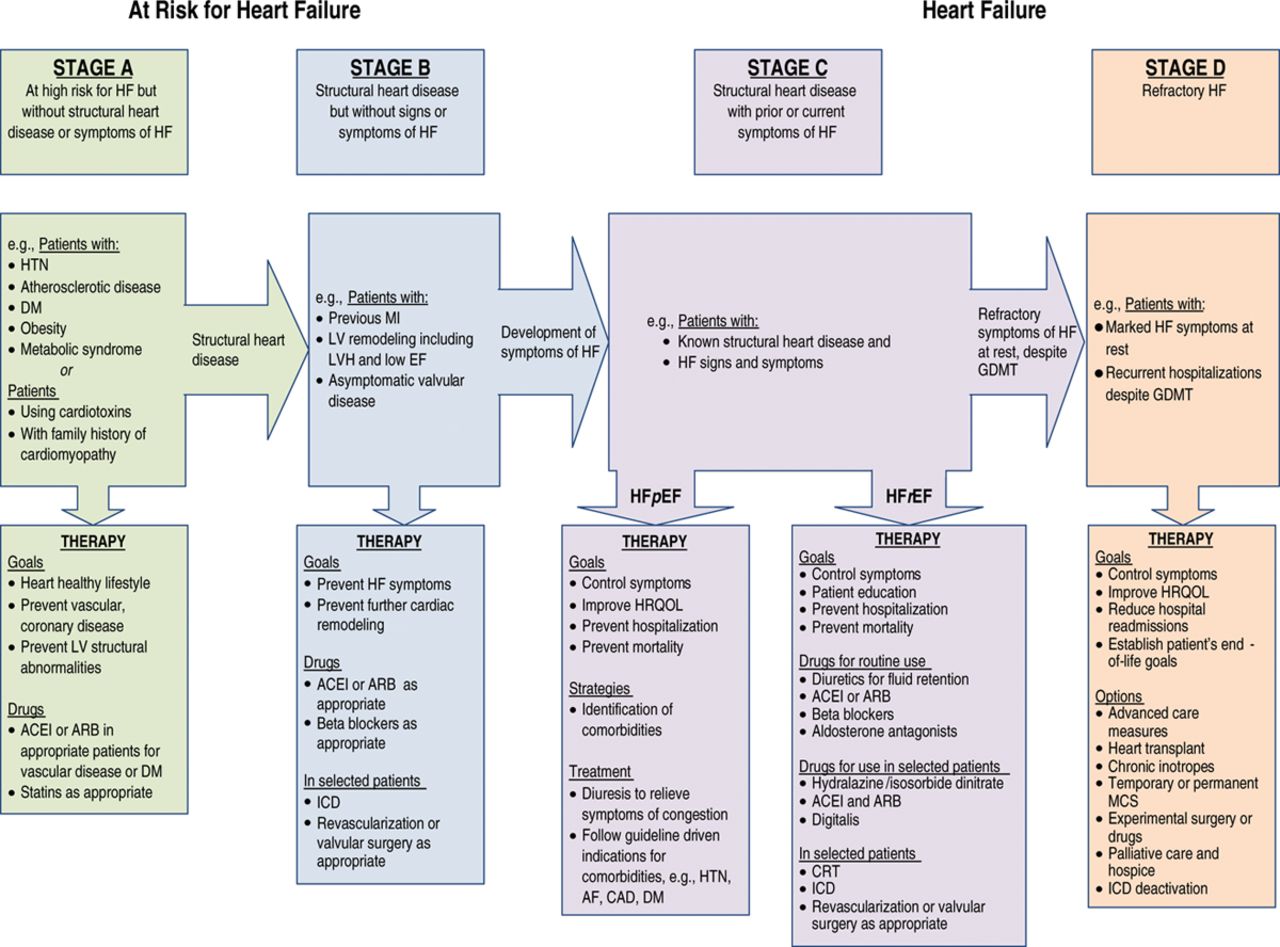


**Fig. 1. Stages in the development of HF and recommended therapy by stage** [1].

- 1. **Exclusion criteria**
- Individuals with type 1 diabetes
- Age <20 years at the time of screening
- HbA1c <7.0% or >9.5% at screening
- FPG >15 mmol/L (270 mg/dL) at screening (Visit 1), confirmed by a repeat test prior to randomization
- Use of a glucagon-like peptide-1 (GLP-1) receptor agonist within 12 weeks prior to the screening visit
- Women of childbearing potential without an effective contraceptive method
- History of gastric surgery, including gastric banding, within 3 years before the screening visit
- History of diabetic ketoacidosis or nonketotic hyperosmolar coma prior to screening.
- Mean systolic blood pressure (BP) >180 mmHg or diastolic BP >95 mmHg after three separate measurements
- History or current symptoms of HF
- Severe anemia, or severe respiratory, hepatic, neurological, or psychiatric disorders; active malignant neoplasms; or any major systemic disease or short life expectancy that would hinder protocol implementation or interpretation of study results
- Aspartate aminotransferase (AST) or alanine aminotransferase (ALT) levels more than three times the upper limit of normal (ULN)
- Total bilirubin >1.5 times the ULN (except for Gilbert syndrome)
- Use of systemic glucocorticoids (excluding topical, ophthalmic, or inhaled forms) for more than 10 consecutive days within 90 days prior to screening
- Participation in another investigational drug trial or use of prohibited therapies within 3 months prior to screening

1. **Major investigation**
   1. **GLS by transcardiac echocardiography**

The standard commercially available cardiac ultrasound machines (Vivid 7 and Vivid E9, General Electric Medical Systems, Milwaukee, WI, USA) will be used to perform resting echocardiograms. Images will be saved in raw data format for offline analysis to assess left ventricular (LV) wall thickness, valvular morphology, chamber volumes, and global 2D strain. LV mass will be measured using standard criteria and normalized to body surface area to obtain the LVMI [3]. LVH will be determined following the recommendations of the American Society of Echocardiography and the European Association of Echocardiography [4]. The modified Simpson’s biplane method will be used to measure the LV ejection fraction (LVEF).

Pulsed wave Doppler recordings of LV inflow will be acquired from the apical four-chamber view with the sample volume placed between the tips of the mitral leaflets. Peak early (E) and late diastolic flow velocities (A), the E/A ratio, and deceleration time of early diastolic flow will be measured. Pulsed wave tissue Doppler will be performed to assess peak early diastolic mitral annular velocity (e’). The ratio of mitral inflow early diastolic velocity to the average e' velocity obtained from the septal and lateral sides of the mitral annulus (E/e’) will be calculated to estimate LV filling pressure, with a value >15 indicating elevated LV filling pressure [5].

For global 2D strain analysis, digital loops will be acquired from standard apical four-chamber, three-chamber, and two-chamber views. The LV endocardial border will be traced at the end-systolic frame. The strain curve will be derived from the gray-scale images using dedicated software (EchoPAC, GE, Vingmed, Norway). Peak strain will be defined as the peak negative value on the strain curve during the cardiac cycle. Peak GLS will be calculated for the entire U-shaped LV myocardium as: LVGLS (%) = [(*L* end-systole − *L* end-diastole) / *L* end-diastole] × 100 [6], where “*L*” represents the length of the entire LV myocardium, considered as one continuous segment. GLS will be averaged across the apical 4-, 3-, and 2-chamber views.

A recent study demonstrated a strong positive correlation between GLS and ejection fraction (EF), particularly in individuals with LV systolic impairment (*r*=0.95; *P*<0.001) [7]. In contrast, LV ejection had a relatively weaker correlation with LV twist and torsion. Another study also showed that semi-automated GLS analysis had a strong correlation with EF, regardless of whether it was performed by experienced readers (*r*=0.98) or less experienced readers (*r*=0.91) (Fig. 2) [8]. The semi-automated GLS measurements exhibited less bias between experienced and less experienced readers (−1.0 ± 13%) compared to EF measurements (−3.5 ± 6.7%). Furthermore, repeated GLS measurements showed higher intra-class correlation (ICC=0.98) compared to EF (ICC=0.89). GLS analysis also takes less time, approximately one minute per patient, whereas biplane EF measurements take twice as long. Thus, semi-automated GLS measurements are faster, less dependent on experience, and more reproducible than conventional EF measurements.

In a recent study involving individuals with advanced chronic kidney disease, GLS was found to be a significant predictor of all-cause mortality [hazard ratio (HR), 1.09; 95% confidence interval (CI), 1.02–1.16; *P*<0.05] and cardiovascular (CV) mortality (HR, 1.16; 95% CI, 1.04–1.30; *P*<0.05), even after adjusting for clinical factors such as LVMI and EF [9]. Notably, GLS had greater predictive power for both all-cause and CV mortality compared to EF. Impaired GLS (>−16%) was associated with a 5.6-fold increased risk of CV mortality, even in individuals with preserved EF. Collectively, these findings suggest that GLS is a valuable measure for reflecting myocardial function.


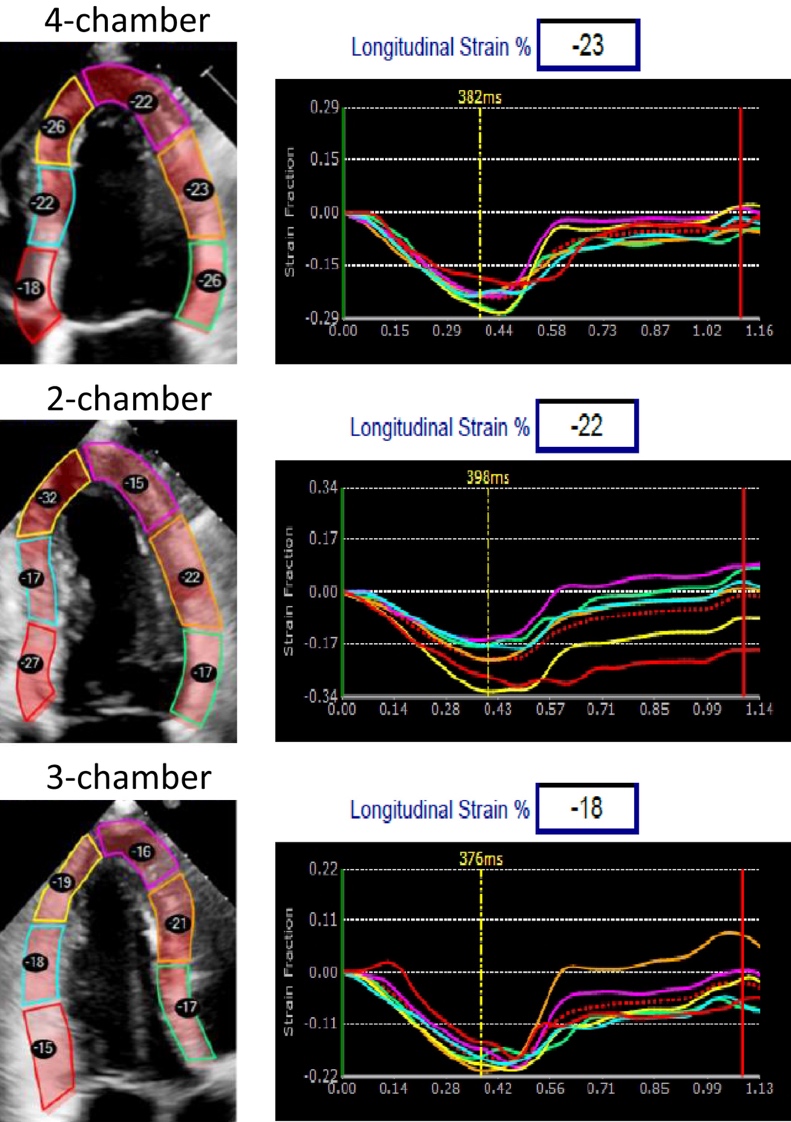


**Fig. 2. Left ventricular global longitudinal strain and volumes.** After the endocardial boundaries are defined by the user, the software generates global longitudinal strain curves throughout the cardiac cycle. These curves quantify myocardial deformation, represented as strain values, across the 4-chamber, 3-chamber, and 2-chamber views [8].

- 1. **Body composition analysis using bioelectrical impedance analysis**

Whole body fat mass and fat percentage are measured using bioelectrical impedance analysis (BIA), a widely used method for estimating body composition by analyzing the impedance generated when a current flows through the body. This noninvasive, simple, and cost-effective technique has evolved from earlier single-frequency systems to the more accurate multifrequency systems used today. To date, body composition data—including whole-body fat mass, fat percentage, and muscle mass—obtained via BIA has been used in over 2,000 studies, including our own, according to a PubMed search conducted on June 15, 2015 [10, 11].

Participants are instructed to avoid smoking, alcohol consumption, and strenuous exercise for 48 hours prior to measurement. After standing on the device platform, age and gender information is entered into the machine. The participant's posture is confirmed, with arms slightly apart from the body and feet placed correctly on the platform, before a supervisor initiates the assessment. The hands are positioned at a 45-degree angle away from the body. The BIA device, such as the X-scan model, uses multiple frequencies (1, 5, 50, 250, 500 kHz, and 1 MHz) to assess intracellular and extracellular fluid levels, as well as total body water content.

Abdominal visceral fat area (VFA) is also estimated using three multifrequency BIA devices (InBody 720; InBody Co., Seoul, Korea), with participants measured in a fasting state on the same day as their blood tests [10].

- 1. **Biomarkers**

Recent studies have identified a new regulatory axis within the renin-angiotensin system (RAS) (Fig. 3). In this axis, angiotensin (1-7) is ultimately produced from either angiotensin I or II through the catalytic activity of angiotensin-converting enzyme 2 (ACE2).


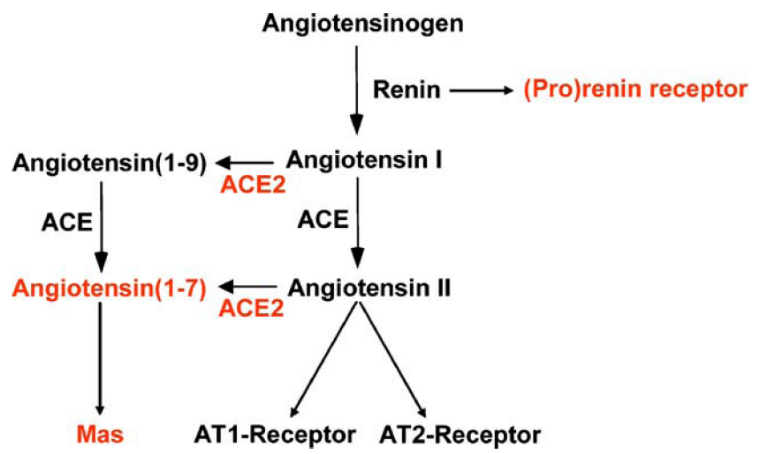


**Fig. 3. Renin-angiotensin system.**

***ACE2***

ACE2 is a monocarboxypeptidase primarily expressed in vascular endothelial cells and renal tubular epithelium. The catalytic domain of ACE2 shares 42% identity with that of angio-converting enzyme (ACE); however, unlike ACE, ACE2 activity is not inhibited by ACE inhibitors. The membrane-bound ectodomain of ACE2 can be cleaved by the metalloproteinase ADAM17, releasing a soluble form of ACE2. Thus, measuring plasma ACE2 reflects the level of soluble ACE2. Soluble ACE2 levels and activity in plasma are elevated in patients with chronic heart failure, cardiac dysfunction, and myocardial infarction. While ACE2 is believed to play a key role in cardiac protection, the exact mechanisms remain unclear.

***Angiotensin (1-7)***

Angiotensin (1-7) is primarily produced from angiotensin II by ACE2, with the heart, brain, and kidneys serving as key sites of its generation. It exerts broad effects on the cardiovascular system, including vasodilation, myocardial protection, antiarrhythmic actions, and antihypertensive effects. Additionally, it may inhibit vascular inflammation and smooth muscle cell proliferation via the Mas receptor. Angiotensin (1-7) is also believed to have beneficial effects on metabolism by reducing insulin resistance

***Pro–B-type natriuretic peptide***

Cardiac natriuretic peptides are released by the heart under physiological conditions and play a key role in maintaining cardiovascular homeostasis. They induce dilation of afferent arterioles and constriction of efferent arterioles in the kidneys, which increases glomerular filtration, promoting natriuresis and diuresis. Additionally, these peptides reduce cardiac preload by shifting intravascular fluid into the interstitial space. Natriuretic peptides also suppress the RAS, inhibiting renin release from the kidneys and aldosterone secretion from the adrenal glands, further enhancing natriuresis and reducing extracellular fluid volume. B-type natriuretic peptide (BNP) belongs to the natriuretic peptide family, which includes atrial natriuretic peptide (ANP) and C-type natriuretic peptide, the latter primarily secreted by the vascular endothelium. ANP and BNP are secreted by cardiac myocytes, with BNP predominantly released by atrial myocytes in a healthy heart. However, in congestive HF, ventricular BNP secretion increases significantly, reflecting greater ventricular stress and severe cardiac dysfunction. In patients with HF, BNP levels can rise by up to 107-fold compared to healthy individuals.

***High-sensitivity C-reactive protein***

Increased levels of the inflammatory biomarker high-sensitivity C-reactive protein (hsCRP) are predictive of cardiovascular events. In a trial involving apparently healthy individuals without hyperlipidemia but with elevated hsCRP, the lipid-lowering drug rosuvastatin significantly reduced hsCRP levels by 37% and lowered the incidence of major cardiovascular events [12]. hsCRP concentrations were measured using enzyme-linked immunosorbent assay kits from BD Biosciences Pharmingen (BD Biosciences Pharmingen, Heidelberg, Germany).

1. **Study outcomes**
   1. **Primary outcome**

- Changes in LVGLS after ertugliflozin treatment in individuals with T2D and pre-HF compared to placebo.
  1. **Secondary outcomes**
- Changes in LVEF
- Changes in LVMI
- Changes in E/e’
- Changes in left atrial volume index
- Changes in LV end-diastolic volume
  1. **Prespecified exploratory outcomes**
- Changes in body weight, body fat mass, and body fat percentage with ertugliflozin
- Whether ertugliflozin reduces microalbuminuria
- Impact of ertugliflozin on ACE2, angiotensin (1-7), pro-BNP, and hsCRP levels
- Cardiovascular benefits of ertugliflozin, including improvement in diastolic dysfunction and activation of the ACE2-angiotensin (1-7) axis
  1. **Safety outcome**
- Adverse events related to ertugliflozin treatment in individuals with T2D and pre-HF

1. **Treatment**
   1. **Intervention and control**

After randomization, patients will undergo a 24-week double-blind treatment period. A total of 102 patients aged 20 years or older will be randomly assigned in a 1:1 ratio to one of the following two groups:

- Ertugliflozin 5 mg once daily
- Placebo (identical in appearance to ertugliflozin) once daily
  1. **Rescue therapy**

The following criteria will trigger alerts: 1) FPG >270 mg/dL (13.3 mmol/L) at the Week 8 visit; 2) FPG >240 mg/dL (11.1 mmol/L) at the Week 16 visit.

Open-label rescue medication(s) to manage hyperglycemia will be administered at the investigator’s discretion, following local standards of care and prescribing practices. Any approved medication, including oral antidiabetic drugs or insulin, may be used to treat hyperglycemia, with the exception of other SGLT2 inhibitors. If glycemic rescue is necessary, the investigational product administered during the randomized, double-blind treatment phase should be continued, and blinding must be maintained until the end of the study.

1. **Sample size calculation**

The primary endpoint of this study is the change in LVGLS from baseline to week 24. The hypothesis is that the ertugliflozin group will demonstrate superiority over the placebo group.

- Null hypothesis (*H₀*): *P₁* = *P₂* ≥ *δ*
- Alternative hypothesis (*H₁*): *P₁* − *P₂* < *δ*

The following equation will be used for sample size estimation:

When *r* = 1,

1. Type Ⅰ error (*α*) = 5% (two-sided, default)
2. Power = 1 − Type Ⅱ error (*β*) = 80% (default)
3. Allocation (*r*) = 1 (default)
4. Mean in the ertugliflozin group (*μ₁*)
5. Mean in the placebo group (*μ₂*)
6. Standard deviation (SD) in the ertugliflozin group (*SD₁*)
7. SD in the placebo group (*SD₂*)
8. Dropout rate (*d*) = 10% (default)

Since no prior interventional studies have evaluated the effect of ertugliflozin on GLS, we referenced studies measuring LV volume. An increase in LV volume has been used as a marker for assessing the impact of drug therapy on heart failure survival [13]. It has been suggested that a 10 mL change in LV volume is clinically significant [14]. This change corresponds to a 15% change in EF in individuals with pre-HF, whose EF typically ranges from 30% to 50%. In heart failure populations, the SD for the mean difference in LV volume has been reported to be 7.5 [15]. For comparison, the EMPA-REG OUTCOME trial showed that empagliflozin reduced heart failure hospitalization by 35% [16], and the CANVAS program found that canagliflozin reduced heart failure hospitalization by 33% [17]. Based on these results, we assumed a 15% baseline GLS in both groups and a 13.2% decrease in GLS in the ertugliflozin group after treatment, with no change in the placebo group. We assumed a standard deviation of 3.5 for both groups, adopting a conservative approach. Considering an 18% dropout rate, the minimum required sample size is 100 participants (60 per group with a 1:1 randomization). Additionally, a recent study demonstrated that improvements in glycemic control (HbA1c reduction from 10.3 ± 2.4% to 8.3 ± 2.0%) over a 12-month period led to a 21% improvement in GLS and a 24% improvement in cardiac function, as estimated by septal e’ [18].

***Real calculation***

1. α = 0.05
2. 1 − *β* = 0.8
3. *r* = 1
4. *μ_1_* = 17
5. *μ_2_* = 15
6. *SD₁* = 3.5
7. *SD₂* = 3.5
8. *d* = 0.18 (18%)

***Number of study participants***

- Group 1: 46
- Group 2: 46
- Total: 92

After considering the dropout rate, the total required number of participants is 102.

**References**

1. Yancy CW, Jessup M, Bozkurt B, Butler J, Casey DE, Jr., Drazner MH, Fonarow GC, Geraci SA, Horwich T, Januzzi JL *et al*: **2013 ACCF/AHA guideline for the management of heart failure: executive summary: a report of the American College of Cardiology Foundation/American Heart Association Task Force on practice guidelines**. *Circulation* 2013, **128**(16):1810-1852.

2. Kosmala W, Jellis CL, Marwick TH: **Exercise limitation associated with asymptomatic left ventricular impairment: analogy with stage B heart failure**. *J Am Coll Cardiol* 2015, **65**(3):257-266.

3. Devereux RB, Alonso DR, Lutas EM, Gottlieb GJ, Campo E, Sachs I, Reichek N: **Echocardiographic assessment of left ventricular hypertrophy: comparison to necropsy findings**. *Am J Cardiol* 1986, **57**(6):450-458.

4. Lang RM, Bierig M, Devereux RB, Flachskampf FA, Foster E, Pellikka PA, Picard MH, Roman MJ, Seward J, Shanewise JS *et al*: **Recommendations for chamber quantification: a report from the American Society of Echocardiography's Guidelines and Standards Committee and the Chamber Quantification Writing Group, developed in conjunction with the European Association of Echocardiography, a branch of the European Society of Cardiology**. *J Am Soc Echocardiogr* 2005, **18**(12):1440-1463.

5. Nagueh SF, Appleton CP, Gillebert TC, Marino PN, Oh JK, Smiseth OA, Waggoner AD, Flachskampf FA, Pellikka PA, Evangelista A: **Recommendations for the evaluation of left ventricular diastolic function by echocardiography**. *J Am Soc Echocardiogr* 2009, **22**(2):107-133.

6. Reisner SA, Lysyansky P, Agmon Y, Mutlak D, Lessick J, Friedman Z: **Global longitudinal strain: a novel index of left ventricular systolic function**. *J Am Soc Echocardiogr* 2004, **17**(6):630-633.

7. Lima MSM, Villarraga HR, Abduch MCD, Lima MF, Cruz C, Sbano JCN, Voos MC, Mathias WJ, Tsutsui JM: **Global Longitudinal Strain or Left Ventricular Twist and Torsion? Which Correlates Best with Ejection Fraction?** *Arq Bras Cardiol* 2017, **109**(1):23-29.

8. Medvedofsky D, Kebed K, Laffin L, Stone J, Addetia K, Lang RM, Mor-Avi V: **Reproducibility and experience dependence of echocardiographic indices of left ventricular function: Side-by-side comparison of global longitudinal strain and ejection fraction**. *Echocardiography* 2017, **34**(3):365-370.

9. Krishnasamy R, Isbel NM, Hawley CM, Pascoe EM, Burrage M, Leano R, Haluska BA, Marwick TH, Stanton T: **Left Ventricular Global Longitudinal Strain (GLS) Is a Superior Predictor of All-Cause and Cardiovascular Mortality When Compared to Ejection Fraction in Advanced Chronic Kidney Disease**. *PLoS One* 2015, **10**(5):e0127044.

10. Lee DH, Park KS, Ahn S, Ku EJ, Jung KY, Kim YJ, Kim KM, Moon JH, Choi SH, Park KS *et al*: **Comparison of Abdominal Visceral Adipose Tissue Area Measured by Computed Tomography with That Estimated by Bioelectrical Impedance Analysis Method in Korean Subjects**. *Nutrients* 2015, **7**(12):10513-10524.

11. Park KS, Lee DH, Lee J, Kim YJ, Jung KY, Kim KM, Kwak SH, Choi SH, Park KS, Jang HC, Lim S: **Comparison between two methods of bioelectrical impedance analyses for accuracy in measuring abdominal visceral fat area**. *J Diabetes Complications* 2016, **30**(2):343-349.

12. Ridker PM, Danielson E, Fonseca FA, Genest J, Gotto AM, Jr., Kastelein JJ, Koenig W, Libby P, Lorenzatti AJ, MacFadyen JG *et al*: **Rosuvastatin to prevent vascular events in men and women with elevated C-reactive protein**. *N Engl J Med* 2008, **359**(21):2195-2207.

13. Konstam MA, Kramer DG, Patel AR, Maron MS, Udelson JE: **Left ventricular remodeling in heart failure: current concepts in clinical significance and assessment**. *JACC Cardiovasc Imaging* 2011, **4**(1):98-108.

14. Grothues F, Smith GC, Moon JC, Bellenger NG, Collins P, Klein HU, Pennell DJ: **Comparison of interstudy reproducibility of cardiovascular magnetic resonance with two-dimensional echocardiography in normal subjects and in patients with heart failure or left ventricular hypertrophy**. *Am J Cardiol* 2002, **90**(1):29-34.

15. Kramer DG, Trikalinos TA, Kent DM, Antonopoulos GV, Konstam MA, Udelson JE: **Quantitative evaluation of drug or device effects on ventricular remodeling as predictors of therapeutic effects on mortality in patients with heart failure and reduced ejection fraction: a meta-analytic approach**. *J Am Coll Cardiol* 2010, **56**(5):392-406.

16. Zinman B, Wanner C, Lachin JM, Fitchett D, Bluhmki E, Hantel S, Mattheus M, Devins T, Johansen OE, Woerle HJ *et al*: **Empagliflozin, Cardiovascular Outcomes, and Mortality in Type 2 Diabetes**. *N Engl J Med* 2015, **373**(22):2117-2128.

17. Neal B, Perkovic V, Mahaffey KW, de Zeeuw D, Fulcher G, Erondu N, Shaw W, Law G, Desai M, Matthews DR, Group CPC: **Canagliflozin and Cardiovascular and Renal Events in Type 2 Diabetes**. *N Engl J Med* 2017, **377**(7):644-657.

18. Leung M, Wong VW, Hudson M, Leung DY: **Impact of Improved Glycemic Control on Cardiac Function in Type 2 Diabetes Mellitus**. *Circ Cardiovasc Imaging* 2016, **9**(3):e003643.

# Measurement of biochemical parameters

Plasma glucose concentration was measured using the glucose oxidase method with a 747 Clinical Chemistry Analyzer (Hitachi, Tokyo, Japan). HbA1c level was measured using a Variant Ⅱ Turbo HPLC Analyzer (Bio-Rad, Hercules, CA, USA) in a laboratory certified by the National Glycohemoglobin Standardization Program Level Ⅱ. Fasting plasma C-peptide and insulin levels were assessed by radioimmunoassay (Linco, St. Louis, MO, USA). Serum AST, ALT, and creatinine levels were analyzed using an Architect Ci8200 analyzer (Abbott Laboratories, Abbott Park, IL, USA). The eGFR was calculated using the creatinine-based Chronic Kidney Disease Epidemiology Collaboration (CKD-EPI) equation. Serum levels of free fatty acids, total cholesterol, triglycerides, high-density lipoprotein cholesterol, and low-density lipoprotein cholesterol levels were measured using a 747 Clinical Chemistry Analyzer (Hitachi, Tokyo, Japan). Urinary albumin concentration was measured using turbidimetry (502X, A&T, Tokyo, Japan), and urinary creatinine was measured using the Jaffe method (Hitachi 7170, Hitachi, Tokyo, Japan). The ratio of urinary protein or albumin to creatinine concentration (mg/g) was used to identify proteinuria and albuminuria, respectively.

To estimate pancreatic β-cell function and insulin resistance, the homeostasis model assessment of insulin resistance (HOMA-IR) and β-cell function (HOMA-β) indices were calculated.

Safety parameters were also monitored, including adverse events such as gastrointestinal discomfort, urinary tract infections, genital infections, ketoacidosis, and hypoglycemia. Hypoglycemic events were recorded based on patient-reported symptoms and self-monitored glucose levels <70 mg/dL (3.9 mmol/L).
